# Therapeutic potential of serotoninergic psychedelic substances in the treatment of Obsessive Compulsive Disorder

**DOI:** 10.1192/j.eurpsy.2022.1659

**Published:** 2022-09-01

**Authors:** J. Rodrigues, O. Nombora, L. Ribeiro

**Affiliations:** Vila Nova de Gaia Hospital Center, Psychiatry And Mental Health Service, Vila Nova de Gaia, Portugal

**Keywords:** obsessive compulsive disorder, serotoninergic psychedelics, 5-HT2 receptor agonism

## Abstract

**Introduction:**

Obsessive Compulsive Disorder (OCD) is a psychiatric disorder associated with suffering and disability. The serotoninergic system is implicated in the neurobiological processes of OCD and serotonin reuptake inhibitors (SRIs) are the first-line treatment. However, clinical improvement after starting SRIs can take long and patients may not fully recover. Meanwhile, recent data suggests that activation of 5-HT receptors may exert a therapeutic action in obsessional symptoms. Some psychedelics are strong 5-HT2 receptor agonists and there is a growing research interest as they can be a promising therapeutic approach to OCD.

**Objectives:**

We aim to provide an overview on the current evidence on the therapeutic potential of serotoninergic psychoactive substances in the treatment of OCD.

**Methods:**

Non-systematic review. Literature search in the PubMed database using the terms psychedelics and obsessive-compulsive disorder.

**Results:**

Although research is currently limited to a few small studies, the ones conducted so far showed clinically meaningful acute reduction of OCD symptoms after treatment with serotoninergic psychoactive drugs, as well as possible longer-lasting benefits, particularly with psilocybin and lysergic acid diethylamide (LSD). Furthermore, substance-assisted psychotherapy with psychedelics has been showing promising results, being suitable for OCD treatment. It is important to add that, to date, studies have indicated relatively good tolerability to these drugs.

**Conclusions:**

These promising early findings highlight the role of psychedelics in OCD treatment and the need for further research into efficacy, therapeutic mechanisms and safety, in order to determine whether these drugs may be worthy options for OCD treatment in the future.

**Disclosure:**

No significant relationships.

